# Claudin-2 expression is upregulated in the ileum of diarrhea predominant irritable bowel syndrome patients

**DOI:** 10.3164/jcbn.16-92

**Published:** 2017-02-16

**Authors:** Haruka Ishimoto, Tadayuki Oshima, Hiroo Sei, Takahisa Yamasaki, Takashi Kondo, Katsuyuki Tozawa, Toshihiko Tomita, Yoshio Ohda, Hirokazu Fukui, Jiro Watari, Hiroto Miwa

**Affiliations:** 1 Division of Gastroenterology, Department of Internal Medicine, Hyogo College of Medicine, 1-1 Mukogawa-cho, Nishinomiya, Hyogo 663-8501, Japan

**Keywords:** claudins, irritable bowel syndrome, tight junction, mast cell, eosinophil

## Abstract

Intestinal epithelial barrier function is impaired in irritable bowel syndrome patients. Claudins are highly expressed in cells with tight junctions and are involved in the intestinal epithelial barrier function. The expression pattern of tight junction proteins in diarrhea-predominant irritable bowel syndrome have not been fully elucidated. We therefore recruited 17 diarrhea-predominant irritable bowel syndrome patients and 20 healthy controls. The expression of the tight junction-related proteins was examined in the ileal, cecal, and rectal mucosa of diarrhea-predominant irritable bowel syndrome patients using real-time PCR and immunofluorescence. Claudin-2 expression was high in the ileum of diarrhea-predominant irritable bowel syndrome patients. Claudin-2 expression was the same in cecum and rectal mucosa of control and diarrhea-predominant irritable bowel syndrome patients. Similarly, the expression of clauidn-1, claudin-7, JAM-A, occludin, and ZO-1 in the ileal, cecal, and rectal mucosa did not change between control and diarrhea-predominant irritable bowel syndrome samples. Infiltration of eosinophil and mast cells in the mucosa of ileum, cecum and rectum was evaluated using immunohistochemical staining and was not affected by diarrhea-predominant irritable bowel syndrome. Claudin-2 was expressed on the apical side of villi and crypts of ileal mucosal epithelial cells. Clauidn-2 expression is upregulated in diarrhea-predominant irritable bowel syndrome patients and may contribute to the pathogenesis of this condition.

## Introduction

Irritable bowel syndrome (IBS) is a common bowel disorder, with a prevalence estimated to be 10% worldwide.^(1,2)^ Recent studies have shown that intestinal permeability is disrupted in a subset of IBS patients.^(2–5)^ Fecal supernatant of diarrhea-predominant IBS patients (IBS-D) increases intestinal permeability,^(6–10)^ implying that soluble mediators might be involved. However, it is not clear whether impaired mucosal permeability affects the small or large intestine. Several studies have suggested that intestinal permeability is high in IBS-D patients.^(3,4,11)^

IBS is characterized by low-grade inflammation of the intestinal mucosa, mast cell and T cell infiltration, and proinflammatory cytokines in the ileocecum and colon.^(12–16)^ Psychological stress and mucosal mast cells contribute to the pathogenesis of IBS, regardless of its subtypes.^(17)^ In particular, stress induces gastrointestinal permeability through corticotrophin-releasing hormone (CRH) signaling, which stimulates the release of intestinal permeability mediators, such tryptase, from mast cells. Increased permeability is blocked by the mast cell stabilizer, lodoxamide or by disodium cromoglycate pretreatment,^(18,19)^ confirming the importance of these cells in intestinal permeability.

Tight junctions (TJs) are important structures of the intestinal barrier. The expression of proteins contributing to these structures is miss-regulated in IBS patients.^(20)^ For example, surface-expressed TJ proteins such as claudins, occludin and junctional adhesion molecule (JAM)-A, as well as the scaffold protein zonula occludens (ZO)-1, which is involved in the control of intestinal permeability, have altered expression in IBS patients. In particular, the colonic mucosa of IBS patients displays low occludin expression.^(7)^ Another report indicates that occludin and claudin-1 protein levels are lower in IBS-D compared to constipation-predominant IBS (IBS-C) or IBS with mixed bowel habits (IBS-M), even though the expression of occludin and claudin-1 is similar in IBS-C, IBS-M and healthy subjects.^(8)^ Although these data indicate changes in the expression of genes encoding for TJ proteins in IBS patients, these studies only investigated specific intestinal regions of the intestines. No systematic study on TJ proteins has been undertaken to date. Consequently, expression of TJ-related factors is thus not fully understood and requires further investigation. We therefore examined the expression of TJ-related genes and inflammatory cell infiltrations in the ileum, cecum and rectum in IBS-D patients.

## Materials and Methods

### Human endoscopic biopsies

Seventeen patients with IBS-D without any macroscopic lesion in the lower gastrointestinal tract detectable with endoscopy and 20 healthy controls were recruited at Hyogo College of Medicine, Japan, between 2015 and 2016. IBS-D was diagnosed using the Japanese version of the Rome III Diagnostic Questionnaire for Irritable Bowel Syndrome.^(21)^ Exclusion criteria for all participants were intake of nonsteroidal anti-inflammatory drugs, corticosteroids, anti-allergic drugs, or other immunosuppressive drugs in the two months before the study, allergies or inflammatory bowel diseases. Endoscopic biopsy samples were taken from the mucosa of the ileum (approximately 5 cm from ileocecal valve), cecum and rectum of IBS-D patients and healthy donors. One biopsy sample was immediately stored in RNAlater (Qiagen, Hilden, Germany), and was maintained at –20°C for expression analysis. Another biopsy sample was fixed with 10% neutral formalin and embedded in paraffin for immunohistochemistry. Patient anonymity was preserved. This study was performed in accordance with the Declaration of Helsinki and was approved by the Ethics Committee/Institutional Review Board of Hyogo College of Medicine, Japan (no. 174). The subjects gave written informed consent prior to the study.

### Quantitative reverse transcription PCR (qRT-PCR)

 Total mRNA was extracted using Trizol reagent according to the manufacturer’s instructions (Invitrogen Life Technologies, Carlsbad, CA). cDNA was synthesized using a high capacity complementary DNA reverse transcription kit (Applied Biosystems, Foster City, CA). qRT-PCR was performed using a PCR master mix and a 7900HT fast real-time PCR system (Applied Biosystems).

TaqMan probes and primers for claudin-1 (accession no. Hs 00221623_m1; Applied Biosystems), claudin-2 (Hs 00252666_s1), claudin-7 (Hs 00600772_m1), JAM-A (Hs 00170991_m1), occludin (Hs 00170162_m1) and ZO-1 (Hs 00268480_m1) were Assay-On Demand^TM^ gene expression products. The GAPDH gene (Hs 02758991_g1) was used as an endogenous control. The thermal cycler conditions were as follows: 2 min at 50°C, 10 min at 95°C, followed by 40 cycles of 15 s at 95°C for denaturing and 1 min at 60°C for annealing/extension. All procedures were repeated in triplicate. Amplification data were analyzed with Sequence Detection System version 2.2 (Applied Biosystems). TheΔΔCT method recommended by the manufacturer was used to compare the relative expression levels.

### Immunohistochemistry of mast cells and eosinophils

Paraffin-embedded sections of 4 µm in thickness of ileum, cecum and rectum were deparaffinized and blocked with 3% hydrogen peroxide and protein blocking solution (X0909; Dako, Carpinteria, CA). Mast cells and eosinophils were stained by incubating sections at room temperature for 30 min in rabbit anti-human CD117, c-kit (1:500; Dako), and 60 min in mouse anti-eosinophil major basic protein (MBP) (MON6008; 1:30; Monosan, Uden, The Netherlands), respectively. Next, slides were incubated with secondary Envision Dual Link System-HRP^®^ (K4063; Dako). Diaminobenzidine was used as the chromogen and was followed by counterstaining with Mayer’s hematoxylin. Mast cells and eosinophils were counted in at least five representative non-overlapping high-power fields at 400× magnification in a blinded manner.

### Immunofluorescence staining of junctional proteins

Paraffin-embedded sections of 4 µm in thickness of the ileum, cecum and rectum were deparaffinized, heated in citrate buffer (pH 7.0) for epitope retrieval, and then blocked with phosphate-buffered saline containing 5.0% bovine serum albumin. The sections were incubated with primary rabbit anti-claudin-2 and mouse anti-occludin antibodies (1:100; ThermoFisher Scientific, Waltham, MA) at room temperature for 60 min. Subsequently, the sections were incubated with Cy3-conjugated goat anti-rabbit IgG (1:200; ThermoFisher Scientific) and Alexa488-conjugated goat anti-mouse IgG (1:200; ThermoFisher Scientific) at room temperature for 30 min and then nuclei were counterstained with 4',6-diamidino-2-phenylindole (DAPI). The sections were then examined under a microscope (Olympus CX41, Tokyo, Japan). ZEN 2012 software (Carl Zeiss, Tokyo, Japan) was used for image processing.

### Statistical analysis

A two-tailed Mann-Whitney *U* test was performed to compare the two groups. All tests were applied two-sided with a significance level of *p*<0.05. GraphPad Prism6 software (GraphPad Software, Inc. La Jolla, CA) was used for statistical analysis.

## Results

### Subjects

Thirty-seven participants, including 17 patients (11 males, 6 females; mean age 54 ± 19 years) with IBS-D, and 20 control subjects (15 males, 5 females; mean age 64 ± 12 years), were considered for this study. All IBS-D patients met the Rome III criteria for IBS-D.^(22)^

### Expression levels of genes encoding TJ-related proteins

**Fig. 1:**
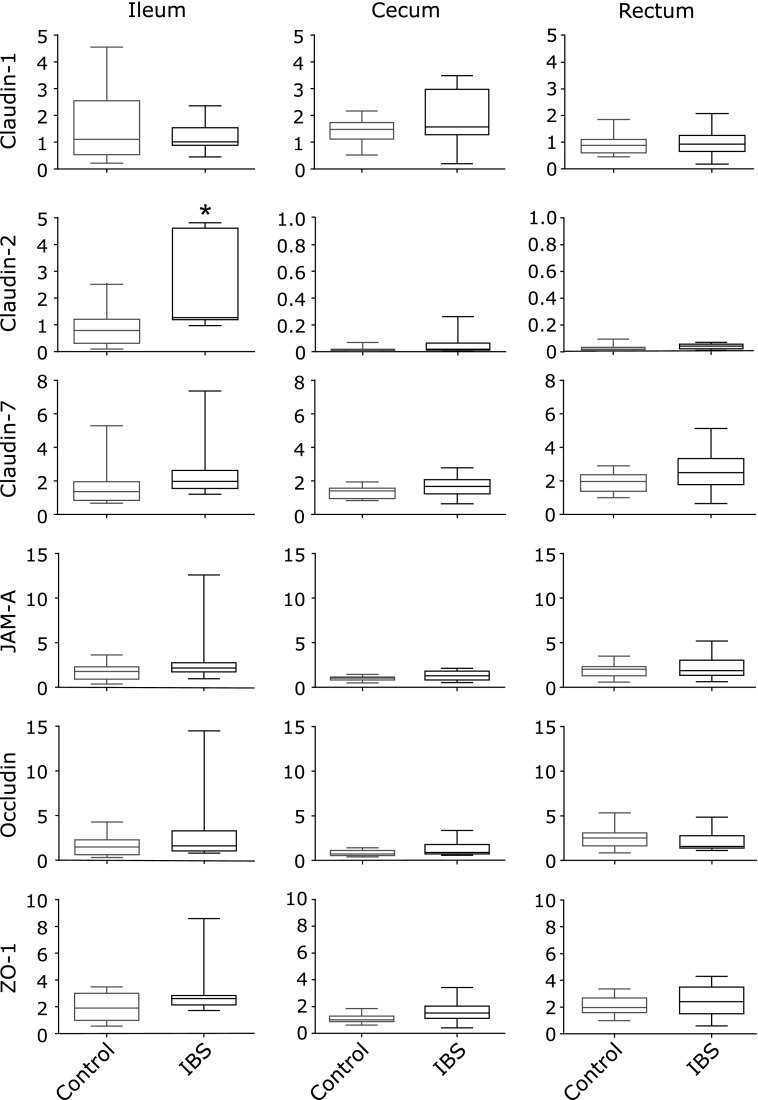
Expression levels of genes encoding TJ-related proteins. Expression levels of mRNA were analyzed using qRT-PCR in ileum, cecum and rectum of healthy donors (control) and IBS-D (IBS) patients. *p<0.05 compared to the relative control.

Expression levels of claudin-1, claudin-2, claudin-7, JAM-A, occludin and ZO-1 were measured in the ileum, cecum and rectum. The expression of claudin-1, claudin-7, JAM-A, occludin and ZO-1 was similar in all intestinal tract and between IBS-D patients and healthy donors. Claudin-2 expression was higher in the ileum compared to cecum and rectum. Furthermore, claudin-2 expression was significantly higher in IBS-D patients when compared with the control group (Fig. 1).

### Eosinophil and mast cell infiltration

**Fig. 2:**
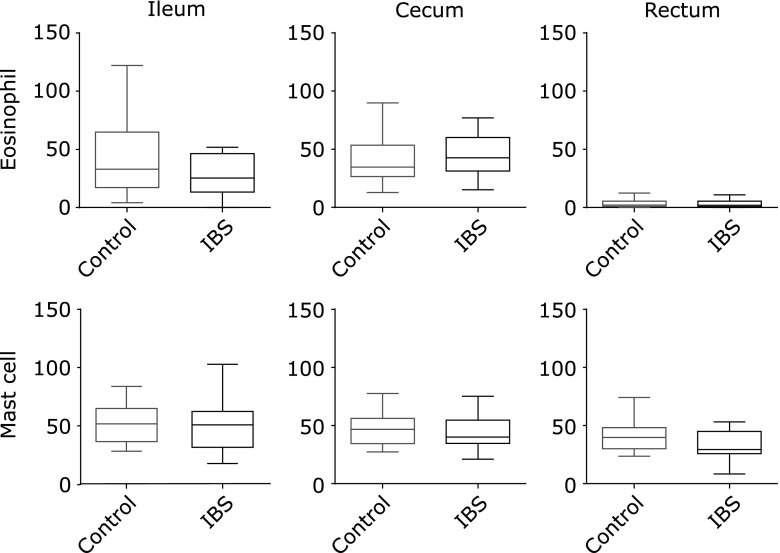
Infiltration of eosinophil and mast cells in controls and IBS-D patients. Infiltrated eosinophil and mast cells were counted in the ileum, cecum and rectum of healthy donors (control) and IBS-D (IBS) patients.

Infiltrated eosinophils and mast cells were counted in the ileum, cecum and rectum. Eosinophil counts were higher in the ileum and cecum compared to the rectum. Mast cell counts were similar in all three intestinal regions. The number of infiltrated eosinophils and mast cells were similar in control groups and IBS-D patients (Fig. 2).

### Expression of claudin-2 and occludin in ileum epithelial cells

**Fig. 3:**
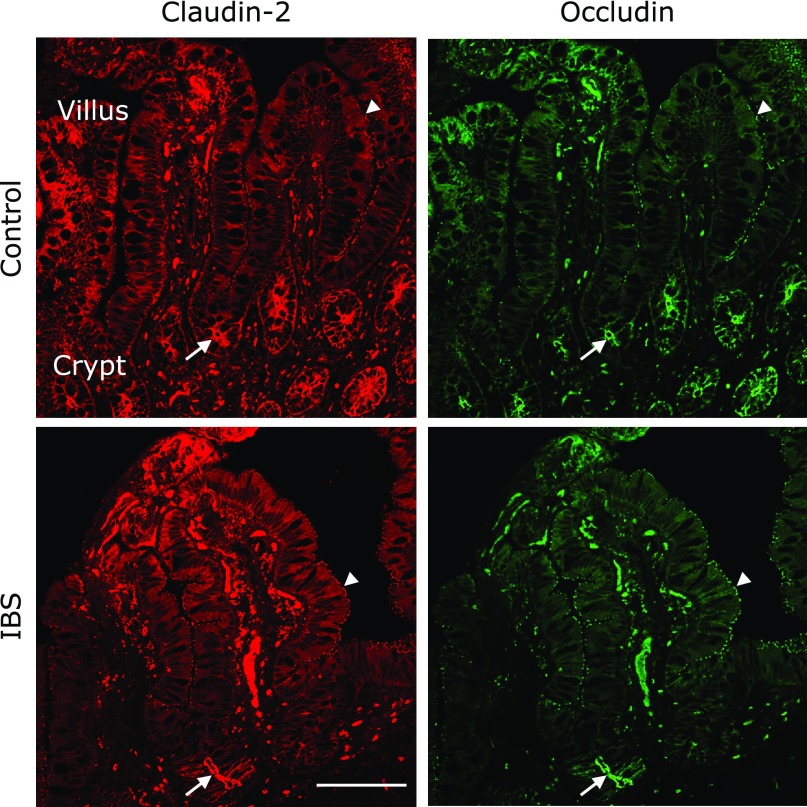
Expression of claudin-2 and occludin in ileum epithelial cells. Apical epithelial surface of crypts and villus cells was positive with claudin-2 and occludin in both controls and IBS-D (IBS) samples. White arrows indicate the location of apical junctional staining. Bar, 100 µm.

As protein quantity itself does not provide information on the effect of TJ proteins on intestinal permeability, influenced instead by their localization,^(20)^ immunofluorescence staining was performed. Immunofluorescence analysis showed that claudin-2 was present in the ileal mucosa, with a strong signal in crypts, and on the apical epithelial surface of the villi. In both cases, it colocalized with occludin. There were no differences in the expression pattern of claudin-2 between control and IBS-D (Fig. 3).

## Discussion

Intestinal epithelial cells maintain a barrier against bacteria, macromolecules and caustic injuries. The loss of functional integrity of this intestinal epithelial barrier leads to gastrointestinal disorders, including IBS.^(3)^ We systematically investigated the expression of TJ-related genes and found that claudin-2 expression was upregulated in the ileal mucosa of IBS-D patients. In contrast, the expression of claudin-1, claudin-7, JAM-A, occludin or ZO-1, as well as cecal and rectal claudin-2 expression were the same in IBS-D patients and the control group. Furthermore, the expression of claudin-2 was highter in the ileum of both IBS-D patiens and healthy donors compared to the respective cecum and rectum samples. As clauidn-2 is a pore-forming TJ-related protein,^(23)^ these data indicate that the upregulation of claudin-2 expression in the ileum may be an important pathophysiological factor related to IBS-D development.

As for the pathophysiology of IBS, multiple factors, including genetic and psychosociologic factors, alterations in the gut microbiome, and changes in immune, motor, and sensory responses may be involved. Although the causes of increased intestinal permeability in IBS are still unclear, these factors regulate mucosal permeability. For example, stress-derived CRH increase the expression of claudin-2, resulting, in turn, in increased intestinal permeability.^(24)^ Application of microbial products induces claudin-2 expression and increases intestinal permeability, indicating that the gut microbiome affects intestinal permeability, potentially leading to the infiltration of inflammatory cells.^(25)^

In this study, the infiltration of eosinophils and mast cells were similar between IBS-D and controls. Eosinophils are potent innate immune cells and are needed to maintain intestinal epithelial homeostasis. Eosinophil-derived soluble mediators induce mast cell activation, resulting in colonic and jejunal epithelial barrier dysfunction.^(19,26)^ Although a previous study reported that the mast cell count in terminal ileal mucosa increases in IBS patients, those authors also showed that the mast cell count is not correlated with the clinical features of this condition.^(16)^ In contrast, mast cell activation in the jejunum of IBS-D patients is correlated with their symptoms.^(27)^ Several reports have shown an increase in the number of mast cells in intestines of IBS-D patients. However, the data are not consistent with ordinal classifications.^(28)^ Sampling criteria and methodological differences may explain the differences between our data and previous reports.^(29)^ Furthermore, as another possibility, inflammatory changes may not be detectable with conventional endoscopically acquired mucosal biopsies. Further studies categorizing the IBS subtypes that show an increased number of mast cells are needed, since mast cell stabilizers can diminish intestinal permeability.^(18)^

The claudin family consists of at least 27 members expressed in a tissue-specific manner.^(30)^ While the expression of most claudins is correlated with the tightness of the epithelial barrier, claudin-2 expression has been shown to result in a decrease in the tightness of epithelial barrier and a consequent increase in paracellular water permeability.^(31–33)^ Claudin-2 expression also increases in inflammatory bowel disease patients.^(34,35)^ IBS-D patients display high claudin-2 protein levels in the jejunum.^(5)^ In our study, claudin-2 immunolocalization showed no differences between IBS-D patients and the control group. Future studies will have to address if claudin-2 expression is correlated with the levels of this protein.

Expression of JAM-A in the colon decreases in IBS-D and IBS-M patients, and it is inversely correlated with the degree of abdominal pain. Furthermore, tryptase increases intestinal permeability and suppresses the expression of JAM-A, claudin-1 and ZO-1 in intestinal epithelial cells *in vitro*.^(36)^ Moreover, expression of ZO-1 and E-cadherin is also significantly lower than healthy controls in the surface epithelium of the IBS-D and IBS-M patients.^(37)^ ZO-1 expression and protein levels decrease in IBS-D patients, and the protein is also redistributed from TJ to the cytoplasm.^(27)^ In this study, the expression of both JAM-A and ZO-1 was the same in IBS-D patients and control patients in the ileum, cecum and rectum. These apparent discrepancies should be clarified by further studies.

The study presented here has several limitations. The sample size was small and to counterbalance this limitation, we recruited homogenous subjects by strictly using the Rome III criteria. The causality between the increased permeability and the low-grade inflammation in the mucosa must also be investigated further. Nevertheless, the regulation of the intestinal barrier likely maintains the intestinal homeostasis and regulates the immune system-mediated inflammation cascade.

In summary, we showed that claudin-2 expression was high in the ileal mucosa, but not in the cecum and rectum in IBS-D patients. Further studies are needed to understand the mechanisms and meaning of claudin-2 upregulation to the pathogenesis of IBS-D.

## Abbreviations

CRH: corticotrophin-releasing hormone
IBS: irritable bowel syndrome
IBS-C: constipation-predominant IBS
IBS-D: diarrhea-predominant IBS
IBS-M: IBS with mixed bowel habits
JAM: junctional adhesion molecule
qRT-PCR: quantitative reverse transcription PCR
TJ: tight junction
